# Correlate the cyanogenic potential and dry matter content of cassava roots and leaves grown in different environments

**DOI:** 10.1038/s41598-023-42425-2

**Published:** 2023-09-16

**Authors:** Emmanuel Oladeji Alamu, Gilbert Alfred Dixon, Michael Adesokan, Busie Maziya-Dixon

**Affiliations:** 1 Food and Nutrition Sciences Laboratory, International Institute of Tropical Agriculture, Southern Africa, Research and Administration Hub (SARAH) Campus, PO Box 310142, 10101 Chelstone, Lusaka Zambia; 2 Food and Nutrition Sciences Laboratory, International Institute of Tropical Agriculture, PMB 5230, Ibadan, Oyo State Nigeria

**Keywords:** Biochemistry, Plant sciences

## Abstract

Cassava (Manihot esculenta Crantz) is an essential stable food crop in Sub-Saharan Africa commonly consumed amongst the low-income communities in Africa. Though cassava roots and leaf have vast economic and commercial benefits, it produces cyanogenic glycosides, which are toxic and most often responsible for the bitter taste of some cassava cultivars. The study evaluates the cassava roots and leaves’ cyanogenic potential and dry matter content of the Genetic Gain Assessment trial grown in a different environment. It establishes the association between the cyanogenic potential (CNP) and the roots and leaves dry matter (DM). Genetic Gain Assessment (GGA) cassava genotypes (N = 400) selected for the Uniform Yield Trial (UYT) breeding stage were planted under IVS (Dry season in Inland Valley Hydromorphic area) and Upland (rain-fed conditions) in two locations of IITA Research Farms, namely; Ibadan (IVS and Upland) and Mokwa (Upland) in Nigeria. The CNP content of cassava leaves in IVS, Mokwa, and Upland ranged from 3.39 to 272.16 mg/100 g, 4.28 to 228.72 mg/100 g, and 13.13 to 127.39 mg/100 g, respectively. However, the respective CNP range in root samples across IVS, Mokwa, and Upland was 0.76–76.31 mg/100 g, 0.94–136.53 mg/100 g, and 2.37–47.11 mg/100 g. Also, the mean ± SD of DM content of leaves were 27.97 ± 3.01%, 28.81 ± 4.01%, and 13.65 ± 3.69%, respectively, in IVS, Mokwa, and Upland, while the root samples had mean ± SD of DM content of 38.09 ± 4.80%, 32.69 ± ,5.93% and 24.63 ± 5.07% respectively. Furthermore, location and genotype had a highly significant effect (p < 0.001) on the CNP and DM of roots and leaves. Also, linear regressions were established between CNP and DM of root and leaf with regression equation; DM-Root = 1.1999*DM-Leaf (r = 0.956) and CNP-Root = 0.29006*CNP-Leaf (r = 0.54). The relationship between the DM (root and leaf) and CNP (root and leaf) could serve as a valuable “inter-prediction” tool for these parameters.

## Introduction

Cassava (*Manihot esculent Crantz)* is a major perennial food crop that provides food for over 800 million people worldwide^1^. It is an energy source in many tropical countries, including Nigeria, and supplies about 70% of daily calories for more than 50 million people^2^. In Nigeria, cassava is considered a smallholder farmers’ favourite because it is available all year round, is tolerant to low soil fertility, and is resistant to diseases and pests^3^. About half a billion people in the tropical regions of Africa, Latin America, and Asia depend heavily on cassava roots for their diets; they are starch-rich (25–30%) but low in other nutrients like protein and vitamins^4^. Compared with other crops, cassava contributes largely to poverty alleviation by generating income for many households in SSA^3^. Though cassava has vast economic and commercial benefits, it produces cyanogenic glycosides; they are poisonous and cause some cassava varieties’ bitter flavour^1^. Cyanogen is a by-product of enzymatic hydrolysis of specific molecules present in cassava, such as linamarin, lostaustralin, and acetone cyanohydrin^5^. Linamarin is synthesized in the leaves through N-hydroxylation of isoleucine and valine and then transported to the roots^6^. It is known to be stored in the vacuoles of the plant and concentrated more in the leaves and root cortex than in the parenchyma of the plant^7^. Damage to cassava flesh during harvesting allows the reaction of Linamarin and linamarase to release acetone cyanohydrin, which, after decomposition, produces cyanide^6,8^. Cassava cultivars are generally categorized as “bitter” or “sweet,” depending on the level of the cyanogenic glycoside. Bitter cassava cultivars have hydrogen cyanide ranging from 15 to 400 mg hydrogen cyanide per kilogram of fresh roots, while sweet cassava has values of 15–50 mg hydrogen cyanide/kg fresh cassava^3^.

Generally, the processing of cassava products tends to reduce the cyanide content significantly. Regrettably, careful processing usually results in the loss of some macro and micronutrients such as proteins, vitamins, and minerals, thereby reducing the product’s nutritional value. Breeding efforts to reduce the level of cyanogenic glycoside is the principal approach. Despite the efficient processing techniques, cyanide exposure from cassava products still poses a significant concern^9^. Conventional breeding has generated cassava cultivars with low to high cyanide but has not provided cultivars with no cyanogenic glycosides^10,11^. In their study, Jorgenson et al.^12^ obtained transgenic cassava plants with more than 99% reduction in cyanide potential and 92% tuber reduction. In IITA, more than one million cassava seedlings were evaluated for low cyanide using the picrate method. A base population of low cyanide cassava was obtained through multiple and continuous selections and recombination of the genotypes with low cyanogenic potential to improve people of low cyanogenic potential and combine high-yielding potential with pest resistance^13^. The Genetic Gain Assessment (GGA) trials are cassava genotypes developed and selected for the Uniform Yield Trial (UYT) breeding stage. At this stage, they were taken into multilocation On-farm practices for nomination for National Coordinated Research Project on Cassava (NCRP) trials by the National Root Crops Research Institute (NRCRI)- an agricultural research institute in Nigeria before the release. Cyanide determination was initially determined by picrate paper methods developed by Mburu et al.^14^. It consisted of placing picrate paper at the entry of a small transparent plastic bottle (5 × 2 cm) containing about 1 g of sample and then 1 mL of phosphate buffer at pH 8. The bottle is closed and left at ambient temperature for 24 h. The change in colour of picrate paper from yellow to chestnut–red will indicate the release of cyanide in the sample and its absorption by picrate paper. Then, the picrate paper will be removed and placed in a test tube containing 5.0 mL water, and the absorbance of the solution will be measured at 510 nm using a spectrophotometer. The picrate leaf method has been used extensively for screening many clones, but the accuracy of the technique is uncompensated. Therefore, a more accurate process, Technicon AutoAnalyzer^15^, was developed to accurately analyze 300 samples daily^13^. However, the auto-analyzer method also has certain limitations with tedious sample preparations and extractions, especially when a more significant number of cultivars are to be evaluated. A rapid method of determining cassava roots’ cyanogenic potential and dry matter while they are still underground is required to monitor the relationship between root age and cyanogenic potential.

The cyanide contents of cassava leaves and roots depend on the roots’ ages and parts and range from 189 to 2466 ppm^16,17^. Older leaves and roots have lower cyanide compared to the younger ones. Root parts closer to the stem end have different cyanide content than those more relative to the cortex. Also, the leaves in the lower part may have more cyanogenic potential than those in the upper part^7^. Hidayat et al.^7^, reported a significant positive correlation between the cyanogenic potential of roots (Y) and leaves (X) among the 45 Indonesian germplasm which has reasonable cyanogenic possibilities; the regression equation was Y = 36.214 + 1.3085X (r = 0.5228). This current study aims to evaluate the cyanogenic potential and dry matter content of the cassava root and leaf of the Genetic Gain Assessment trial growing in a different environment and establish the association between the cyanogenic and dry matter of the roots and leaf. This study’s findings will significantly benefit the cassava breeding program to select low cyanide genotypes using either the cyanogenic potential or dry matter of the leaves to determine the roots at different maturity periods of the plant.

## Materials and methods

### Genetic materials and field establishment

Four hundred cassava genotypes were planted under IVS (Dry season in Inland Valley Hydromorphic area) and Upland (rain-fed conditions) trials in 2 locations of IITA Research Farms, Ibadan (IVS and Upland) and Mokwa (Upland) in Nigeria during the 2006 and 2007 planting seasons. The climate data of the 2 locations are presented in Supplementary Table S1. An Augmented Completely Randomized Design (ACRD) with three checks, TME 1, 91/02324, and 30572, was used for the IVS and Upland trials, respectively. Planting was done on ridges (30 cm high and 1 m apart) as plots. The IVS trial consisted of one ridge and five plants per plot with 0.5 m spacing between plants and 1 m between ridges. However, for the upland trial, each plot consisted of one ridge and ten plants per plot spaced 0.5 m between plants and 1 m between ridges. There were no fertilizers or herbicides applied to both trials. Manual weeding was done as necessary.

This research field study protocols on cassava were reviewed and approved by the International Institute of Tropical Agriculture Internal Research Review Board (IITA-IRB), ensuring compliance with the relevant institutional and national guidelines and legislation (IITA-IRB-Policy-June2016.pdf).

### Sampling and sample preparation

Sampling and sample preparations follow the method Alamu et al.^18^ described. Five plants per genotype were harvested 12 months after planting (MAP). Three cassava roots of different sizes were selected per genotype randomly and labelled appropriately. The cassava roots were washed with tap water and air-dried. The roots were peeled with a stainless-steel knife and rinsed in deionized water. Each root was quartered by dividing longitudinally into two sections, and the opposite sections were selected and cut into smaller pieces and packed into a Whirl pack bag for subsequent analysis. Before laboratory analysis, cassava leaves were collected from the field, washed, air-dried, homogenized, and packaged.

### Determination of dry matter

Ten grams of the raw root and powdered leaves were weighed in a pre-weighed aluminium can and baked for 16 h at 105 °C in an air convectional oven (Memmert UN 55, GmbH) to achieve constant weight. The dry matter content was estimated as the difference between the mass before drying and the mass loss on drying^19^.

### Determination of cyanogenic potential (CNP)

Each root was homogenized using a laboratory blender with 250 mL of 0.1 M orthophosphoric acid. Leaves were also cut and homogenized for cassava. The homogenate was centrifuged, and extract taken from the supernatant was taken as the extract; 0.1 mL of the enzyme was added to 0.6 ml of the extract. The 3.4 mL of the acetate buffer (pH 4.5) was added and mixed. Following this, 0.6 mL of colourant and 0.2 mL of 0.5% chloramines-T were added to allow the colour to develop fully, and the mixture was left to stand for 15 min. The absorbance value was measured at 605 nm compared to a blank with all the same chemicals added but with 0.1 mL of phosphate buffer instead of KCN^20^.

### Statistical analysis

Descriptive, ANOVA, LSD mean separation, Agglomerate Hierarchical Cluster(AHC) analysis, and Pearson correlation analysis using XLSTAT statistical and data analysis solution. New York, USA https://www.xlstat.com/en)^21^.

## Results and discussion

### Assessment of CNP and DM contents of GGA cassava genotypes (leaf and root) from the different growing environments

**Figure 1:**
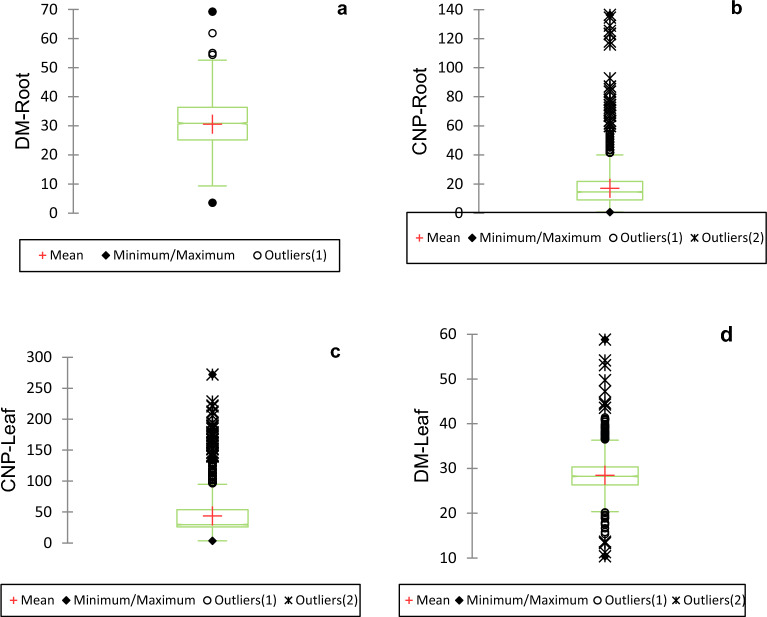
Box plots of cyanogenic potential (CNP) and Dry matter (DM) contents of genetic gain assessment (GGA) cassava genotypes (leaf and root). (**a**) Box plot for DM-Root; (**b**) box plot for CNP-Root; (**c**) box plot for CNP-Leaf; (**d**) box plot for DM-Leaf.

Table 1 and Fig. 1 summarise descriptive statistics for CNP and DM contents of cassava roots and leaves across the three planting environments. CNP content of cassava leaves in IVS, Mokwa, and Upland ranged from 3.39 to 272.16 mg/100 g, 4.28 to 228.72 mg/100 g, and 13.13 to 127.39 mg/100 g, respectively. However, the respective CNP range in root samples across IVS, Mokwa, and Upland was 0.76–76.31 mg/100 g, 0.94–136.53 mg/100 g, and 2.37–47.11 mg/100 g. These values agree with previously published studies^18,22^. Also, the varieties in IVS and Mokwa with a minimum CNP concentration of 0.94 mg/100 g respectively in the root are within the World Health Organization limit of 1 mg/100 g (10 ppm)^23^. The mean ± SD of DM content of foliage were 27.97 ± 3.01%, 28.81 ± 4.01%, and 13.65 ± 3.69%, respectively, in IVS, Mokwa, and Upland, while the root samples had mean ± SD of DM content of 38.09 ± 4.80%, 32.69 ± 5.93% and 24.63 ± 5.07% in the respective locations. The DM of the root samples analyzed in this study is consistent with the values reported by Kundy et al.^24^, Oly-Alawuba and Agbugbaeruleke^25^, and Alamu et al.^18^. For cassava foliage, samples from IVS had the highest CNP with a mean value of 68.14 mg/100 g, while Upland had the least CNP with an average value of 26.44 mg/100 g. On the other hand, DM content was highest in foliage samples from Mokwa with a mean value of 28.81% and most diminutive in representatives from IVS with an average of 27.97%. However, for root samples, CNP was highest in samples from Mokwa (21.86 mg/100 g) and lowest in samples from IVS (12.43 mg/100 g); meanwhile, for DM content, it was highest in IVS (38.09%) and most deficient in Upland (24.63%). Cassava has been classified based on the CNP levels as sweet or nontoxic, with CNP levels below 50 ppm, while levels between 50 and 100 ppm are considered moderately harmful. Bitter cassava has CNP levels above 100 pm and is classified as poisonous and unsafe for consumption. The levels of CNP in the roots in this current study are primarily below 100 pm for the locations studied. In general, the average CNP was consistently higher in foliage than in roots across the three environments, which concurs with the study by Burns et al.^22^. This is a result of cyanide synthesis, which starts from the shoot apex and the leaves of the cassava plant and is then transported to the roots, thereby making cassava leaves have higher cyanide concentration than roots^12,26^. Burns et al.^22^ also reported that leaves from plants experiencing water stress tend to have high cyanide content, consistent with the observed high CNP of leaves samples from IVS.

**Table 1:** Descriptive statistics of cyanogenic potential (CNP) and dry matter (DM) contents of genetic gain assessment (GGA) cassava genotypes (leaf and root) from 3 different growing locations.

Parameter (fresh weight basis)	Trial-Location	# of observations	Minimum	Maximum	1st quartile	Median	3rd quartile	Mean	Variance (n-1)	Standard deviation (n-1)	Standard error of the mean
CNP-Leaf (mg/100 g)	IVS-Ibadan	798	3.39	272.16	44.59	60.00	86.57	68.14	1234.59	35.14	1.26
CNP-Root (mg/100 g)	IVS-Ibadan	798	0.76	76.31	5.63	9.63	16.64	12.43	94.54	9.72	0.34
DM-Leaf (%)	IVS-Ibadan	798	10.40	47.25	26.36	27.99	29.64	27.97	9.03	3.01	0.11
DM-Root (%)	IVS-Ibadan	798	18.85	69.31	35.19	38.47	41.09	38.09	23.00	4.80	0.17
CNP-Leaf (mg/100 g)	Mokwa	1489	4.28	228.72	28.62	41.37	59.40	48.13	884.51	29.74	0.80
CNP-Root (mg/100 g)	Upland-Mokwa	1489	0.94	136.53	11.63	18.11	28.97	21.86	245.98	15.68	0.41
DM-Leaf (%)	Upland-Mokwa	1489	11.29	58.83	26.25	28.56	30.86	28.81	16.11	4.01	0.11
DM-Root (%)	Upland-Mokwa	1489	12.51	47.59	29.43	33.38	36.77	32.69	35.15	5.93	0.15
CNP-Leaf (mg/100 g)	Upland-Ibadan	1533	13.13	127.39	24.73	26.48	28.13	26.44	19.21	4.38	0.11
CNP-Root (mg/100 g)	Upland-Ibadan	1533	2.37	47.11	9.46	13.78	18.19	15.01	52.79	7.27	0.19
DM-Leaf (%)	Upland-Ibadan	1533	10.40	58.83	26.32	28.25	30.35	28.50	13.65	3.69	0.08
DM-Root (%)	Upland-Ibadan	1533	3.57	42.22	21.44	24.95	28.12	24.63	25.72	5.07	0.13

### Effect of genotypes and growing location on the CNP and DM contents of GGA cassava root and leaf

Table 2 shows the effect of genotype and growing location of the CNP and DM content of GGA cassava root and foliage. In contrast, Supplementary Table S2 shows the means of the parameters by genotypes across the three locations. Both site and genotype had a highly significant effect (p < 0.001) on the CNP of root and leaves and DM of root and leaves. However, the location did not significantly affect the mean DM of leaf samples from Mokwa and Upland. This could result from the similar environmental conditions of these two locations. The significant effects of genotype and location have been previously reported^18,22,27^. The effects of the environment and genotypes respond differently to changes in environmental conditions, which could be attributed to different climatic conditions such as rainfall, temperature, soil pH, and soil fertility. This significant GxE supports the importance of testing cassava across multiple locations to characterize genotype performance accurately^28^. Also,^29^ confirmed the effects of genotypes and genotype-environment interaction on cassava genotypes grown at different locations in Tanzania.

**Table 2:** Cyanogenic potential (CNP) and dry matter (DM) contents of genetic gain assessment (GGA) cassava genotypes (leaf and root) by location.

Location	CNP-Root (mg/100 g)	DM-Root (%)	CNP-Leaf (mg/100 g)	DM-Leaf (%)
IVS	12.469 c	37.962 a	67.038 a	27.996 b
Mokwa	22.002 a	32.542 b	48.174 b	28.712 a
Upland	15.524 b	24.642 c	26.032 c	28.516 a
Pr > F(Genotype)	< 0.0001	< 0.0001	< 0.0001	< 0.0001
Pr > F(Location)	< 0.0001	< 0.0001	< 0.0001	< 0.0001

### Cluster analysis of genetic gain assessment (GGA) cassava genotypes (leaf and root) CNP and DM contents

**Figure 2:**
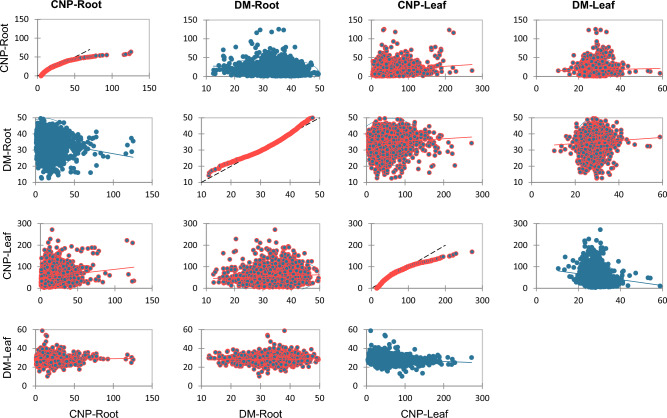
Q–Q plots of correlation between cyanogenic potential (CNP) and dry matter (DM) contents of genetic gain assessment (GGA) cassava genotypes (leaf and root).

Table 3 and Fig. 2 show the hierarchical cluster analysis of the 400 GGA cassava genotypes. Cluster analysis classifies multivariate datasets into subgroups based on population similarities^30^. In 2021, 224 cassava genotypes were grouped into 3 clusters based on their carotenoids, vitamin C, cyanide, and dry matter compositions^18^ similar to what we obtained. They showed in their study that cluster 1 had 56.6% of the total population, while 2 and 3 had 42.9 and 3.57%, respectively. In this study, the dendrogram shows the association of the genotypes based on similarities in their cyanide and dry matter content across different locations. Different genotypes were put into various groups. The samples were divided into two clusters, with cluster 2 showing greater values for all parameters and models other than the leaf DM. Cluster 1 has 1510 populations, more than four times as much as Cluster 2, which only has a population of 461.

**Table 3:** Cluster analysis of genetic gain assessment (GGA) cassava genotypes (leaf and root) growing in 3 locations using cyanogenic potential (CNP) and dry matter (DM) contents.

Cluster	1	2				
Number of objects by cluster	1510	461				
Sum of weights	1510	461				
Within-cluster variance	489.384	1172.206				
Minimum distance to the centroid	1.256	2.651				
Average distance to the centroid	19.786	27.363				
Maximum distance to the centroid	108.551	168.311				

Description of the cluster centroids						
Cluster	CNP-Root (mg/100 g)	DM-Root (%)	CNP-Leaf (mg/100 g)	DM-Leaf (%)	Sum of weights	Within-cluster variance
1	17.12	34.54	41.48	28.63	1510.00	489.38
2	20.10	35.85	103.94	27.74	461.00	1172.21

Moreover, all the check samples were found in cluster 2. It implies that all the genotypes in cluster 2 with superior dry matter (DM) roots could interest the breeder. The clustering could assist the breeders in identifying genotypes with good dry matter and low cyanide content immediately.

### Pearson correlation between CNP (root and leaf) and DM (root and leaf)

Figure 2 and Table 4 present Pearson’s correlation statistics of CNP and DM parameters. The analysis measured the linear correlation between dry matter and cyanide composition to indicate their direction within the sample populations^31^. A strong positive linear relationship exists between CNP-leaf, CNP-root (r = 0.158), DM-leaf, and DM-root (r = 0.056). Also, CNP-roots had a negative correlation (p ≤ 0.001, r = − 0.194) with DM-root, while DM-Leaf and CNP-Leaf had a negative correlation (p ≤ 0.001, r = − 0.15), respectively. Furthermore, DM-root had a significant negative correlation (r = − 0.194) with CNP-root at p ≤ 0.001, whereas it had a positive significant (p ≤ 0.05, r = 0.082) correlation with CNP-leaf. The relationship between CNP-leaf and CNP roots had the highest positive correlation of 0.158, indicating that the root’s cyanide content could be drawn from the leaf.

**Table 4:** Pearson correlation coefficient of cyanogenic potential (CNP) and dry matter (DM) contents of genetic gain assessment (GGA) cassava genotypes (leaf and root).

Parameters	CNP-Root	DM-Root	CNP-Leaf	DM-Leaf
CNP-Root	1.000			
DM-Root	− 0.194***	1.000		
CNP-Leaf	0.158***	0.082**	1.000	
DM-Leaf	0.030	0.056*	− 0.147***	1.000

On the other hand, the dry matter of the leaf shows a negative trend in the CNP of the roots. Nweke et al.^10,11^ also reported a strong negative relationship between CNP roots by establishing a positive correlation with moisture which could be linked to the solubility of hydrogen cyanide in water. The current study also confirms the negative correlation d DM-roots and CNP roots, which agrees with the previous study. A positive correlation between traits means they could be bred together in breeding programs, and negatively correlated traits would be challenging to combine^32^. The relationship between the CNP-leaf and CNP roots could be valuable in their “inter-prediction” in cassava breeding programmes.

### Linear regression of CNP and DM in cassava root and leaf

**Figure 3:**
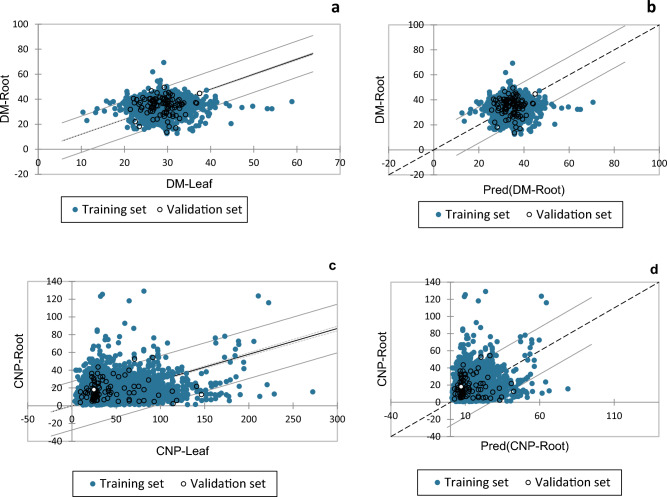
(**a**) Regression of DM-Root by DM-Leaf; (**b**) regression of predicted (DM-root)–actual DM-Root; (**c**) regression of CNP-Root by CNP-Leaf; (**d**) regression of predicted (CNP-Root)–CNP-Root.

Figure 3 and Table 5 describe the regression between the CNP and DM of the roots and leaves of cassava. The regression equations of DM and CNP were validated using an independent set of samples by predicting the DM of roots using the DM of the leaf and then the CNP of the roots from the CNP of the leaf, respectively. The coefficient of determination (R^2^) was 0.95 with a Root Mean Square Error of 7.83 for DM, while R^2^ of 0.54 was obtained for CNP with an RMSE of 13.86. Results showed that the prediction of DM of roots from the leaves gives more accuracy than CNP of roots from the CNP leaf. The regression equations for DM and CNP of the roots and leaves are as follows:

1 $${\text{DM - Root }} = { 1}.{1999 }*{\text{ DM - Leaf }}({\text{R}}^{{2}} = \, 0.{956})$$

2 $${\text{CNP - Root }} = \, 0.{29}00{6 }*{\text{ CNP - Leaf }}\left( {{\text{R}}^{{2}} = \, 0.{59}} \right).$$

**Table 5:** Goodness of fit statistics (DM-Root) using DM-Leaf and CNP root using CNP-Leaf.

Statistic	DM-Root by DM-Leaf		CNP-Root by CNP-Leaf	
	Training set	Validation set	Training set	Validation set
Observations	1940	100	3551	100
Sum of weights	1940	100	3551	100
DF	1939	99	3550	99
R^2^	0.95	–	0.59	–
Adjusted R^2^	0.95	–	0.59	–
MSE	54.51	59.95	193.17	191.81
RMSE	7.383	7.743	13.89	13.85

*MSE* mean square error, *RMSE* root mean square error.

The regression models could be used as a rapid prediction tool for DM and CNP of the roots using their equivalent content in the leaves. However, Hidayat et al.^7^ reported a significant linear regression of CNP in the leaves and the roots of 99 genotypes from Indonesia, with a regression coefficient (R^2^) of 0.52. The regression coefficient in the study showed slightly improved performance compared to those previously reported. This could be due to the large datasets used and significant variations among the genotypes. However, we planned to apply the equations to predict the DM root and CNP root from the DM leaf and CNP leaf, respectively, using a new set of genotypes and improving the models’ accuracy and robustness.

## Conclusions

Genetic Gain Assessment (GGA) trials which have attained the advanced breeding stage and are next to take for on-farm multilocation trials, have been purposively selected to establish the relationship between CNP-roots vs CNP-leaf and DM-roots vs DM-leaf. This study has shown that location and genotype had a highly significant effect on the CNP of roots and leaves and DM of roots and leaves. The maximum CNP in the roots and leaves were found in genotypes 99/0110 and MM97/0016, respectively, while the genotypes 93/0658 and 01/1412 had the maximum DM in roots and leaves, respectively. Minimum CNP and DM in the roots were observed in genotypes 92/1154 and 99/214, respectively. Linear regressions were established between cyanide and dry matter of roots and leaves, providing an indirect determination of cassava roots from the leaves, which could be relevant to monitor the build-up of cyanide in cassava as its ages. Generally, the findings from this study will significantly benefit the cassava breeding program to select low cyanide genotypes using either the cyanogenic potential or dry matter of the leaves to determine the roots at different maturity periods of the plant.

### Supplementary Information

Supplementary Table 1.

Supplementary Table 2.

## Data Availability

The data associated with this study are presented in the supplementary tables, and upon request, the corresponding author will provide additional data.
