# Supplementary material for: Correlate the cyanogenic potential and dry matter content of cassava roots and leaves grown in different environments
Source: Sci Rep. 2023 Sep 16;13:15382. doi: 10.1038/s41598-023-42425-2 (PMC10505158; doi:10.1038/s41598-023-42425-2)
Supplement: Supplementary file 1 — Supplementary Table 1. [file 41598_2023_42425_MOESM1_ESM.docx]

|  |  |  |  |  |  |  |  |  |  |  |  |  |  |
| --- | --- | --- | --- | --- | --- | --- | --- | --- | --- | --- | --- | --- | --- |
| **Location** |  | **Jan** | **Feb** | **Mar** | **April** | **May** | **Jun** | **July** | **Aug** | **Set** | **Oct** | **Nov** | **Dec** |
| **Ibadan (forest-savanna transition zone;7° 38'N, 3° 89'E**) | Rainfall | 0.3″ | 0.8″ | 1.8″ | 3.6″ | 5.4″ | 7.6″ | 7.4″ | 7.1″ | 9.1″ | 6.0″ | 1.2″ | 0.3'' |
|  | Temp. | 79°F | 81°F | 82°F | 81°F | 80°F | 78°F | 76°F | 76°F | 77°F | 78°F | 79°F | 79°F |
|  |  | **Jan** | **Feb** | **Mar** | **April** | **May** | **Jun** | **July** | **Aug** | **Set** | **Oct** | **Nov** | **Dec** |
| **Mokwa (Southern Guinea savanna zone 9° 28'N, 5° 05'E.)** | Rainfall | 0.0″ | 0.2″ | 0.6″ | 1.7″ | 3.0″ | 3.7″ | 4.9″ | 5.9″ | 6.2″ | 2.8″ | 0.3″ | 0.0″ |
|  | Temp. | 77°F | 82°F | 85°F | 86°F | 83°F | 81°F | 79°F | 78°F | 79°F | 80°F | 79°F | 77°F |

Supplementary Table 1: Climate data on the growing locations
