# Supplementary material for: Correlate the cyanogenic potential and dry matter content of cassava roots and leaves grown in different environments
Source: Sci Rep. 2023 Sep 16;13:15382. doi: 10.1038/s41598-023-42425-2 (PMC10505158; doi:10.1038/s41598-023-42425-2)
Supplement: Supplementary file 2 — Supplementary Table 2. [file 41598_2023_42425_MOESM2_ESM.docx]

| Supplementary Table 2: Means of Cyanide and Dry matter contents by genotypes | | | | |
| --- | --- | --- | --- | --- |
|  | CNP-Root | DM-Root | CNP-Leaf | DM-Leaf |
| 84/00516(4X) | 27.377 cde | 36.817 abc | 61.008 b | 34.657 a |
| 01/0098 | 35.334 bcd | 36.193 abc | 60.946 b | 29.157 bcd |
| 96/0035 | 28.032 cde | 35.320 abc | 68.097 b | 28.857 bcd |
| 00/0350 | 22.192 defghi | 36.873 abc | 49.227 b | 30.502 abcd |
| 00/0345 | 23.007 defghi | 37.331 ab | 54.908 b | 28.719 bcd |
| ATU | 18.592 defghi | 37.009 abc | 76.193 ab | 28.587 bcd |
| 91/00106(4X) | 31.114 cde | 32.404 abc | 52.911 b | 29.951 abcd |
| 00/0364 | 19.292 defghi | 34.078 abc | 75.052 ab | 29.252 bcd |
| 00/0351 | 18.385 defghi | 35.818 abc | 58.647 b | 29.189 bcd |
| 88/00279 | 40.167 abcd | 35.973 abc | 52.027 b | 28.389 bcd |
| 01/0103 | 22.326 defghi | 38.330 a | 42.904 b | 31.655 abc |
| 92/0509 | 34.925 bcd | 34.278 abc | 53.822 b | 28.519 bcd |
| 89/02195 | 27.143 defg | 31.043 abc | 72.724 b | 29.507 abcd |
| 99/6015 | 33.972 bcde | 32.921 abc | 58.028 b | 28.567 bcd |
| 91/0033(3X) | 25.694 defgh | 37.210 abc | 49.912 b | 28.389 bcd |
| AKINRINADE | 27.355 cde | 34.876 abc | 74.245 ab | 28.081 bcd |
| 93/0614(3x) | 29.272 cde | 35.482 abc | 49.610 b | 28.389 bcd |
| 95/0896 | 24.015 defghi | 33.502 abc | 51.026 b | 28.984 bcd |
| 00/0354 | 15.279 efghi | 39.311 a | 56.893 b | 28.686 bcd |
| 93/0568(4X) | 31.398 cde | 33.399 abc | 43.109 b | 31.949 abc |
| 93/0602(4X) | 20.828 defghi | 35.379 abc | 44.454 b | 31.107 abc |
| 94/0018 | 16.147 efghi | 35.013 abc | 49.372 b | 30.886 abc |
| 01/1335 | 34.940 bcd | 33.600 abc | 44.352 b | 29.878 abcd |
| 91/002629(3X) | 23.306 defghi | 37.778 ab | 48.573 b | 28.389 bcd |
| 94/0330 | 26.890 defg | 31.418 abc | 52.621 b | 29.526 abcd |
| M98/0036 | 32.126 bcde | 28.792 abcd | 78.922 ab | 29.354 bcd |
| 91/00106(4x) | 21.129 defghi | 37.555 ab | 49.874 b | 28.389 bcd |
| 93/0832(4X) | 38.221 abcd | 19.545 d | 80.149 ab | 30.505 abcd |
| 96/1551 | 13.632 efghi | 36.383 abc | 65.610 b | 28.911 bcd |
| 93/0739(3x) | 25.676 defgh | 36.923 abc | 47.601 b | 28.389 bcd |
| 30572(4X) | 31.090 cde | 35.042 abc | 35.063 b | 31.172 abc |
| M85/00046 | 24.467 defghi | 33.496 abc | 77.395 ab | 28.207 bcd |
| LAPAI-1 | 21.719 defghi | 35.099 abc | 83.584 ab | 27.906 bcd |
| 92/1154(3X) | 23.593 defghi | 36.220 abc | 94.879 ab | 27.266 bcd |
| MM97/1307 | 29.288 cde | 26.246 cd | 100.252 ab | 29.555 abcd |
| 01/1551 | 23.790 defghi | 34.670 abc | 41.933 b | 30.273 abcd |
| 87/00038 | 24.069 defghi | 34.580 abc | 50.813 b | 28.389 bcd |
| 99/3133 | 14.443 efghi | 33.629 abc | 62.853 b | 29.439 abcd |
| M94/0192 | 23.112 defghi | 31.537 abc | 66.134 b | 28.670 bcd |
| 91/00420 | 20.055 defghi | 32.426 abc | 60.276 b | 28.847 bcd |
| 96/1800 | 13.176 efghi | 35.607 abc | 67.361 b | 28.873 bcd |
| 91/00153(3x) | 19.556 defghi | 36.598 abc | 50.408 b | 28.389 bcd |
| 93/0509(4X) | 57.179 ab | 30.977 abc | 45.512 b | 33.329 ab |
| 93/0509(4x) | 29.266 cde | 34.156 abc | 49.168 b | 28.389 bcd |
| 93/0598(3x) | 24.695 defghi | 35.828 abc | 47.477 b | 28.389 bcd |
| 99/3151 | 20.278 defghi | 37.669 ab | 57.500 b | 27.839 bcd |
| 93/0568(4x) | 34.708 bcde | 34.281 abc | 47.946 b | 28.389 bcd |
| O87/00611 | 22.145 defghi | 37.417 ab | 31.632 b | 30.048 abcd |
| 01/1635 | 23.858 defghi | 32.562 abc | 46.231 b | 29.711 abcd |
| 97/4580 | 26.862 defg | 34.445 abc | 56.558 b | 27.916 bcd |
| 96/1317 | 21.536 defghi | 31.564 abc | 51.321 b | 29.454 abcd |
| 95/0645 | 14.969 efghi | 34.411 abc | 49.833 b | 29.670 abcd |
| 91/0061(3x) | 26.560 defgh | 33.929 abc | 48.972 b | 28.389 bcd |
| 97/4779 | 31.854 cde | 30.429 abc | 58.198 b | 28.466 bcd |
| 91/00090(3x) | 23.813 defghi | 34.477 abc | 49.045 b | 28.389 bcd |
| 30040 | 12.125 efghi | 34.550 abc | 66.985 b | 29.240 bcd |
| 91/00262(3X) | 26.903 defg | 35.897 abc | 35.558 b | 29.313 bcd |
| 96/1632 | 19.147 defghi | 38.482 a | 33.536 b | 30.217 abcd |
| 99/0111 | 25.332 defgh | 31.474 abc | 50.209 b | 28.923 bcd |
| 01/1172 | 34.943 bcd | 32.473 abc | 54.574 b | 28.200 bcd |
| 93/0681(4x) | 21.083 defghi | 34.187 abc | 50.362 b | 28.389 bcd |
| M98/0068 | 19.344 defghi | 30.837 abc | 67.404 b | 28.986 bcd |
| Akinrinade | 14.198 efghi | 41.217 a | 96.252 ab | 27.827 bcd |
| 97/4763 | 27.188 def | 32.816 abc | 53.640 b | 28.257 bcd |
| 01/1331 | 36.234 abcd | 22.945 cd | 51.530 b | 30.838 abc |
| 01/0090 | 20.562 defghi | 34.717 abc | 37.462 b | 31.121 abc |
| 91/00458 | 20.035 defghi | 35.313 abc | 39.604 b | 29.775 abcd |
| M98/0004 | 22.405 defghi | 28.187 bcd | 79.952 ab | 29.063 bcd |
| 97/2205 | 17.683 defghi | 36.522 abc | 69.118 b | 27.551 bcd |
| 97/4766 | 28.461 cde | 34.050 abc | 49.965 b | 27.991 bcd |
| 93/0591(4X) | 44.770 abcd | 33.822 abc | 29.119 b | 29.944 abcd |
| 91/00255(3x) | 18.965 defghi | 35.000 abc | 49.606 b | 28.389 bcd |
| 96/1672 | 18.865 defghi | 35.427 abc | 47.061 b | 28.481 bcd |
| K95/0562 | 25.869 defgh | 27.402 cd | 69.083 b | 29.021 bcd |
| 91934(4X) | 27.443 cde | 31.849 abc | 67.924 b | 28.000 bcd |
| ABBEY IFE | 12.469 efghi | 41.513 a | 90.814 ab | 28.082 bcd |
| 30555(4x) | 18.510 defghi | 35.106 abc | 49.356 b | 28.389 bcd |
| CB-10(80411) | 22.445 defghi | 23.592 cd | 74.311 ab | 29.510 abcd |
| RB92/0099 | 12.893 efghi | 32.279 abc | 62.828 b | 29.685 abcd |
| CB5-10(80411) | 37.140 abcd | 31.070 abc | 45.486 b | 29.337 bcd |
| 97/0211 | 10.320 efghi | 34.747 abc | 58.333 b | 29.306 bcd |
| 89/00585 | 21.255 defghi | 33.368 abc | 49.895 b | 28.389 bcd |
| 93/0796(3x) | 17.249 efghi | 35.263 abc | 49.744 b | 28.389 bcd |
| 98/0406 | 19.031 defghi | 30.634 abc | 63.704 b | 28.710 bcd |
| 94/0069 | 20.551 defghi | 32.024 abc | 48.728 b | 29.161 bcd |
| 96/0304 | 24.088 defghi | 35.657 abc | 49.332 b | 27.653 bcd |
| MM96/5280 | 9.073 efghi | 34.641 abc | 62.967 b | 29.342 bcd |
| 93/0114 | 18.044 defghi | 33.926 abc | 42.148 b | 30.788 abc |
| 93/0638(4X) | 51.626 abc | 29.587 abcd | 45.100 b | 30.103 abcd |
| 82/00061 | 32.804 bcde | 36.917 abc | 18.346 b | 28.843 bcd |
| MH95/0414 | 8.731 efghi | 36.527 abc | 62.215 b | 28.854 bcd |
| 92/0110 | 10.346 efghi | 37.690 ab | 49.555 b | 29.121 bcd |
| 96/0191 | 16.185 efghi | 36.969 abc | 79.945 ab | 27.048 bcd |
| 2ND AGRIC | 17.805 defghi | 40.036 a | 53.792 b | 27.362 bcd |
| 93/0665(3x) | 16.618 efghi | 36.682 abc | 47.924 b | 28.389 bcd |
| 97/1170 | 11.647 efghi | 38.415 a | 48.589 b | 28.994 bcd |
| 91/00089(3x) | 26.542 defgh | 32.647 abc | 47.579 b | 28.389 bcd |
| 94/0006 | 15.283 efghi | 31.616 abc | 52.056 b | 29.675 abcd |
| OLEKANGA | 13.694 efghi | 31.439 abc | 72.373 b | 29.280 bcd |
| 97/0270 | 10.547 efghi | 37.045 abc | 51.609 b | 28.708 bcd |
| 91/00262(3x) | 26.619 defgh | 32.691 abc | 47.113 b | 28.389 bcd |
| 84/00316(4X) | 44.891 abcd | 30.793 abc | 36.646 b | 32.631 abc |
| Bagiwawa | 18.007 defghi | 36.515 abc | 98.995 ab | 24.745 d |
| 91/00416 | 14.676 efghi | 32.989 abc | 54.184 b | 28.648 bcd |
| 88/02090 | 15.080 efghi | 36.500 abc | 48.710 b | 28.389 bcd |
| 91/00143(3X) | 15.546 efghi | 38.010 ab | 26.575 b | 31.019 abc |
| 93/0739(3X) | 18.182 defghi | 30.702 abc | 56.536 b | 28.758 bcd |
| 85/00680 | 23.836 defghi | 31.430 abc | 50.201 b | 28.389 bcd |
| 98/2226 | 22.322 defghi | 31.944 abc | 70.227 b | 27.618 bcd |
| 91/00082(3x) | 24.632 defghi | 30.509 abc | 51.825 b | 28.389 bcd |
| 81/01623(4x) | 29.769 cde | 30.969 abc | 48.790 b | 28.389 bcd |
| 93/0265 | 22.918 defghi | 26.197 cd | 51.467 b | 29.429 abcd |
| 89/00568(4x) | 13.838 efghi | 37.131 abc | 47.856 b | 28.389 bcd |
| 91/00090(3X) | 16.767 efghi | 35.165 abc | 44.371 b | 28.665 bcd |
| 96/0860 | 13.491 efghi | 38.857 a | 36.537 b | 29.757 abcd |
| 91/01730(4X) | 63.618 a | 28.034 bcd | 139.659 a | 27.849 bcd |
| 99/6069 | 10.275 efghi | 35.326 abc | 49.457 b | 29.087 bcd |
| 42025x81/01623(4x) | 23.858 defghi | 31.549 abc | 48.702 b | 28.389 bcd |
| TOKUNBO | 17.401 efghi | 36.120 abc | 48.692 b | 27.965 bcd |
| 93/0007 | 23.588 defghi | 35.373 abc | 48.009 b | 27.293 bcd |
| 01/1097 | 16.327 efghi | 36.071 abc | 33.761 b | 29.565 abcd |
| 99/6076 | 15.459 efghi | 34.893 abc | 71.881 b | 27.317 bcd |
| 88/02343 | 13.833 efghi | 36.279 abc | 48.028 b | 28.389 bcd |
| 95/0104 | 22.733 defghi | 30.730 abc | 43.004 b | 29.713 abcd |
| 91/02327(4X) | 22.146 defghi | 33.666 abc | 19.147 b | 29.889 abcd |
| 96/0016 | 9.369 efghi | 37.815 ab | 59.622 b | 28.324 bcd |
| 90/3073 | 15.950 efghi | 35.736 abc | 46.793 b | 28.389 bcd |
| 91/00453 | 12.327 efghi | 35.358 abc | 60.592 b | 28.189 bcd |
| 82/00661 | 15.287 efghi | 35.024 abc | 43.973 b | 28.894 bcd |
| 87/00018-42(4X) | 20.927 defghi | 26.714 cd | 57.358 b | 28.939 bcd |
| 93/0832(4x) | 29.289 cde | 31.660 abc | 45.797 b | 28.389 bcd |
| 96/0619 | 15.802 efghi | 33.637 abc | 52.512 b | 28.335 bcd |
| 93/0796(3X) | 17.284 efghi | 35.288 abc | 48.975 b | 27.974 bcd |
| 82/00058(4X) | 42.413 abcd | 26.280 cd | 43.072 b | 30.401 abcd |
| 99/0554 | 18.132 defghi | 38.728 a | 45.024 b | 27.945 bcd |
| 90/01058 | 20.036 defghi | 33.695 abc | 37.779 b | 29.095 bcd |
| 92/0057(4X) | 17.586 defghi | 34.609 abc | 16.664 b | 30.901 abc |
| Z95/0432 | 15.086 efghi | 29.768 abcd | 57.996 b | 29.145 bcd |
| AKINKINADE | 16.809 efghi | 34.494 abc | 46.844 b | 28.389 bcd |
| 01/0034 | 18.299 defghi | 33.598 abc | 41.388 b | 28.996 bcd |
| 30572 | 17.513 efghi | 32.743 abc | 45.301 b | 28.770 bcd |
| 40764 | 13.604 efghi | 30.934 abc | 62.618 b | 28.738 bcd |
| 93/0824(4X) | 23.634 defghi | 29.944 abc | 38.029 b | 31.946 abc |
| 96/1039 | 20.023 defghi | 30.422 abc | 45.905 b | 29.398 abcd |
| 93/0560(3X) | 16.779 efghi | 34.858 abc | 26.748 b | 29.922 abcd |
| 96/0595 | 10.653 efghi | 31.771 abc | 62.526 b | 29.013 bcd |
| 91934 | 29.804 cde | 32.055 abc | 49.102 b | 27.572 bcd |
| 30555P3-2 | 23.883 defghi | 36.934 abc | 31.572 b | 28.244 bcd |
| 95/0460 | 15.703 efghi | 29.596 abcd | 51.592 b | 29.272 bcd |
| 91/00033(3x) | 13.037 efghi | 37.536 ab | 45.852 b | 28.389 bcd |
| 99/0313 | 13.150 efghi | 29.960 abc | 50.286 b | 30.327 abcd |
| 00/0346 | 21.911 defghi | 37.266 ab | 43.732 b | 27.347 bcd |
| 97/0335 | 27.014 defg | 32.683 abc | 44.399 b | 28.172 bcd |
| 30555(4X) | 16.838 efghi | 34.302 abc | 21.232 b | 30.686 abcd |
| 94/0239 | 13.766 efghi | 31.642 abc | 46.798 b | 29.971 abcd |
| 00/0338 | 10.581 efghi | 37.625 ab | 39.194 b | 29.626 abcd |
| 93/0658(3X) | 18.750 defghi | 32.854 abc | 47.594 b | 28.339 bcd |
| 98/0510 | 21.750 defghi | 31.756 abc | 48.273 b | 28.313 bcd |
| 99/0119 | 20.418 defghi | 32.473 abc | 57.588 b | 27.027 bcd |
| 01/1649 | 27.898 cde | 24.894 cd | 46.209 b | 29.337 bcd |
| 01/1646 | 13.749 efghi | 32.166 abc | 48.001 b | 29.048 bcd |
| 42025 X 81/01623(4X) | 37.394 abcd | 29.631 abcd | 29.086 b | 30.293 abcd |
| BAGIWAWA(4X) | 17.468 efghi | 34.223 abc | 66.983 b | 24.748 d |
| M98/0115 | 11.564 efghi | 34.551 abc | 45.885 b | 29.220 bcd |
| 96/0023 | 21.924 defghi | 28.194 bcd | 49.505 b | 28.642 bcd |
| 96/0409 | 17.739 defghi | 33.082 abc | 49.271 b | 28.025 bcd |
| 96/0986 | 11.561 efghi | 32.865 abc | 49.727 b | 28.884 bcd |
| 99/0503 | 18.894 defghi | 29.314 abcd | 52.884 b | 28.393 bcd |
| 96/1708 | 17.405 efghi | 31.557 abc | 62.619 b | 27.852 bcd |
| 91/00078 | 11.917 efghi | 36.851 abc | 19.822 b | 35.046 a |
| 92B/00068 | 14.580 efghi | 34.693 abc | 69.285 b | 26.601 cd |
| BAGIWAWA | 22.348 defghi | 39.419 a | 39.217 b | 27.320 bcd |
| Z97/0002 | 12.861 efghi | 35.852 abc | 24.666 b | 31.929 abc |
| 93/0638(4x) | 17.425 efghi | 32.975 abc | 45.931 b | 28.389 bcd |
| 96/0590 | 5.922 ghi | 34.161 abc | 51.877 b | 28.980 bcd |
| 99/6016 | 23.236 defghi | 36.532 abc | 44.595 b | 26.457 cd |
| 95/0423 | 13.022 efghi | 33.466 abc | 60.527 b | 27.969 bcd |
| 96/1089A | 9.540 efghi | 33.950 abc | 47.018 b | 29.390 abcd |
| 01/0040 | 13.015 efghi | 34.421 abc | 44.863 b | 28.761 bcd |
| 01/0046 | 22.851 defghi | 35.052 abc | 40.206 b | 27.868 bcd |
| M98/0078 | 15.394 efghi | 32.204 abc | 71.868 b | 27.601 bcd |
| 91/00143(4x) | 18.542 defghi | 31.149 abc | 48.863 b | 28.389 bcd |
| M98/0057 | 9.187 efghi | 37.227 ab | 46.749 b | 28.484 bcd |
| MM96/3665 | 22.014 defghi | 33.208 abc | 48.471 b | 27.294 bcd |
| 96/0160 | 12.810 efghi | 33.772 abc | 63.984 b | 27.805 bcd |
| 91/00417 | 19.259 defghi | 34.418 abc | 42.433 b | 28.299 bcd |
| Z97/0299 | 14.559 efghi | 35.976 abc | 31.381 b | 29.123 bcd |
| 95/0967 | 27.486 cde | 30.969 abc | 38.361 b | 28.662 bcd |
| 99/3073 | 9.722 efghi | 35.764 abc | 38.976 b | 30.234 abcd |
| AMALA | 14.448 efghi | 33.719 abc | 78.624 ab | 26.722 cd |
| 01/0093 | 25.486 defgh | 32.131 abc | 34.495 b | 28.488 bcd |
| 91/00078(3X) | 21.824 defghi | 35.966 abc | 40.190 b | 27.713 bcd |
| 96/0603 | 19.886 defghi | 31.940 abc | 43.829 b | 28.443 bcd |
| M94/0583 | 12.532 efghi | 38.233 ab | 52.051 b | 27.057 bcd |
| 30555 | 15.210 efghi | 33.319 abc | 34.135 b | 29.864 abcd |
| 01/0131 | 11.846 efghi | 33.069 abc | 43.442 b | 29.812 abcd |
| 84/00136(4x) | 22.041 defghi | 29.517 abcd | 48.785 b | 28.389 bcd |
| 91/02322 | 21.599 defghi | 29.561 abcd | 54.633 b | 28.135 bcd |
| 30001(4X) | 25.311 defghi | 28.606 bcd | 32.994 b | 31.909 abc |
| 81/01623(4X) | 33.492 bcde | 31.388 abc | 46.870 b | 27.634 bcd |
| 93/0681(4X) | 18.923 defghi | 31.385 abc | 36.606 b | 29.609 abcd |
| 97/0255 | 9.506 efghi | 34.150 abc | 57.871 b | 28.343 bcd |
| 91/00455 | 14.035 efghi | 32.316 abc | 59.893 b | 27.879 bcd |
| 00/0378 | 9.169 efghi | 35.911 abc | 64.294 b | 27.615 bcd |
| 89/00003-1(4X) | 20.359 defghi | 27.741 bcd | 46.296 b | 29.328 bcd |
| 93/0528(4X) | 16.302 efghi | 32.387 abc | 30.441 b | 30.317 abcd |
| 98/2132 | 22.136 defghi | 28.204 bcd | 62.276 b | 27.868 bcd |
| 99/7533 | 21.481 defghi | 33.353 abc | 42.296 b | 28.214 bcd |
| 99/1702 | 7.566 efghi | 32.368 abc | 48.055 b | 29.898 abcd |
| 91/00142(4x) | 21.993 defghi | 28.423 bcd | 49.494 b | 28.389 bcd |
| 99/1734 | 17.381 efghi | 32.357 abc | 34.451 b | 29.360 bcd |
| 96/0102 | 14.825 efghi | 33.495 abc | 56.173 b | 27.420 bcd |
| 01/0169 | 20.631 defghi | 26.421 cd | 42.699 b | 30.587 abcd |
| 92/0326(4X) | 16.538 efghi | 28.378 bcd | 54.196 b | 28.480 bcd |
| 99/1903 | 11.865 efghi | 34.566 abc | 44.193 b | 28.699 bcd |
| 96/0523 | 26.303 defgh | 33.013 abc | 45.014 b | 27.196 bcd |
| 91/00033(3X) | 12.295 efghi | 36.494 abc | 46.896 b | 28.190 bcd |
| 93/0560(3x) | 31.572 cde | 24.341 cd | 48.027 b | 28.389 bcd |
| 93/0639(3x) | 20.035 defghi | 30.418 abc | 47.207 b | 28.389 bcd |
| 95/0279 | 13.391 efghi | 33.304 abc | 44.428 b | 28.612 bcd |
| 89/00003-1(3x) | 25.187 defghi | 26.985 cd | 48.142 b | 28.389 bcd |
| 97/3200 | 26.826 defgh | 27.876 bcd | 73.110 b | 26.896 bcd |
| 87/00018(4X) | 17.387 efghi | 32.185 abc | 58.556 b | 26.960 bcd |
| 00/0355 | 10.174 efghi | 34.341 abc | 42.013 b | 29.530 abcd |
| W4092 | 14.972 efghi | 32.592 abc | 52.883 b | 27.791 bcd |
| 00/0363 | 11.242 efghi | 35.590 abc | 49.457 b | 28.030 bcd |
| 97/0162 | 18.941 defghi | 30.121 abc | 67.383 b | 27.342 bcd |
| 90/01718 | 13.354 efghi | 32.336 abc | 39.068 b | 29.953 abcd |
| 91/02324 | 21.323 defghi | 30.275 abc | 34.022 b | 29.663 abcd |
| 99/6070 | 18.266 defghi | 33.237 abc | 29.148 b | 28.899 bcd |
| 99/3033 | 13.992 efghi | 33.140 abc | 20.240 b | 31.913 abc |
| 99/6067 | 20.983 defghi | 30.001 abc | 102.416 ab | 25.906 cd |
| 93/0266 | 21.068 defghi | 32.503 abc | 43.490 b | 28.139 bcd |
| 91/0006(3X) | 18.017 defghi | 29.502 abcd | 49.994 b | 28.389 bcd |
| 94/0026 | 11.641 efghi | 32.562 abc | 79.134 ab | 27.731 bcd |
| 97/4769 | 13.093 efghi | 34.850 abc | 50.974 b | 27.488 bcd |
| 98/0034 | 13.167 efghi | 35.161 abc | 60.945 b | 25.393 cd |
| 88/112-7(3x) | 16.493 efghi | 29.964 abc | 50.398 b | 28.389 bcd |
| I00/0049 | 20.154 defghi | 32.153 abc | 45.684 b | 28.181 bcd |
| 91/00078(3x) | 15.204 efghi | 32.155 abc | 47.147 b | 28.389 bcd |
| 30572(chk) | 16.116 efghi | 32.376 abc | 47.038 b | 28.348 bcd |
| 93/0386 | 14.549 efghi | 34.722 abc | 37.273 b | 28.542 bcd |
| 96/1314 | 1.478 i | 40.484 a | 40.003 b | 29.239 bcd |
| 95/0902 | 9.925 efghi | 34.816 abc | 39.512 b | 29.385 abcd |
| 92/0427 | 17.903 defghi | 28.813 abcd | 56.848 b | 28.278 bcd |
| 91/00153(3X) | 9.856 efghi | 34.598 abc | 98.975 ab | 26.915 bcd |
| 91/00438 | 18.489 defghi | 31.895 abc | 48.653 b | 27.843 bcd |
| 01/1423 | 13.884 efghi | 31.963 abc | 35.598 b | 31.058 abc |
| 96/0249 | 24.416 defghi | 32.309 abc | 44.128 b | 27.592 bcd |
| ALICELOCAL | 15.596 efghi | 29.966 abc | 50.433 b | 28.389 bcd |
| 89/00963 | 12.428 efghi | 26.854 cd | 81.758 ab | 28.555 bcd |
| 99/6012 | 26.341 defgh | 29.510 abcd | 41.633 b | 28.458 bcd |
| 98/0505 | 23.724 defghi | 28.190 bcd | 83.589 ab | 26.503 cd |
| TME 778 | 12.547 efghi | 35.930 abc | 29.058 b | 29.186 bcd |
| 95/0306 | 9.649 efghi | 36.087 abc | 52.733 b | 27.586 bcd |
| 95/0248 | 11.471 efghi | 31.622 abc | 45.256 b | 29.747 abcd |
| 2nd Agric | 11.451 efghi | 36.620 abc | 55.948 b | 26.600 cd |
| 89/00250 | 20.942 defghi | 28.833 abcd | 47.712 b | 28.389 bcd |
| 92/0067(4x) | 19.165 defghi | 30.490 abc | 46.128 b | 28.389 bcd |
| 01/1224 | 19.687 defghi | 31.025 abc | 49.577 b | 27.717 bcd |
| 01/1560 | 10.008 efghi | 24.916 cd | 56.901 b | 30.766 abc |
| 91/02163 | 11.304 efghi | 32.742 abc | 43.254 b | 29.315 bcd |
| 95/0041 | 23.147 defghi | 29.179 abcd | 47.344 b | 28.245 bcd |
| 91/00700(3X) | 11.929 efghi | 30.962 abc | 42.426 b | 38.819 a |
| 86/1(4X) | 24.245 defghi | 21.645 cd | 34.095 b | 31.573 abc |
| 94/0263 | 22.194 defghi | 31.331 abc | 46.638 b | 27.836 bcd |
| 01/1231 | 13.871 efghi | 35.200 abc | 25.920 b | 28.903 bcd |
| 30572(4x) | 22.140 defghi | 27.568 bcd | 47.234 b | 28.389 bcd |
| 90/00099 | 16.619 efghi | 33.550 abc | 32.428 b | 28.588 bcd |
| 96/0596 | 8.852 efghi | 34.816 abc | 62.085 b | 27.465 bcd |
| MM96/1871 | 7.878 efghi | 32.961 abc | 42.443 b | 30.289 abcd |
| 00/0388 | 13.283 efghi | 38.730 a | 41.714 b | 27.945 bcd |
| 82/00058(4x) | 31.126 cde | 22.505 cd | 46.397 b | 28.389 bcd |
| 91/00089(3X) | 22.955 defghi | 33.765 abc | 36.247 b | 27.829 bcd |
| 96/0097 | 16.131 efghi | 28.821 abcd | 59.364 b | 28.215 bcd |
| 92/0342 | 13.214 efghi | 31.483 abc | 70.766 b | 27.581 bcd |
| 94/0237 | 6.371 fghi | 35.351 abc | 57.888 b | 27.702 bcd |
| 92/1029(3x) | 17.309 efghi | 31.611 abc | 44.205 b | 28.389 bcd |
| 93/0647(4x) | 16.987 efghi | 29.813 abcd | 48.334 b | 28.389 bcd |
| 99/0621 | 10.231 efghi | 34.162 abc | 43.315 b | 28.801 bcd |
| 96/1630 | 13.940 efghi | 31.795 abc | 68.516 b | 27.112 bcd |
| 01/1413 | 14.213 efghi | 24.614 cd | 51.586 b | 29.063 bcd |
| 95/0211 | 10.872 efghi | 30.201 abc | 46.988 b | 29.893 abcd |
| 01/0045 | 2.975 i | 36.822 abc | 31.099 b | 29.999 abcd |
| 93/0591(4x) | 18.249 defghi | 29.600 abcd | 47.039 b | 28.389 bcd |
| 95/0528 | 12.122 efghi | 34.407 abc | 39.132 b | 28.683 bcd |
| MM96/JW1 | 4.659 hi | 34.989 abc | 46.331 b | 28.574 bcd |
| 92/0342(4X) | 25.425 defgh | 29.771 abcd | 53.387 b | 26.019 cd |
| 93/0658(3x) | 16.575 efghi | 30.376 abc | 47.212 b | 28.389 bcd |
| 81/00110 | 21.412 defghi | 32.320 abc | 43.121 b | 27.782 bcd |
| K95/0725 | 18.564 defghi | 30.621 abc | 29.099 b | 29.541 abcd |
| 87/00018-42(4x) | 18.220 defghi | 29.386 abcd | 47.228 b | 28.389 bcd |
| 90/1554 | 15.471 efghi | 28.993 abcd | 50.595 b | 28.389 bcd |
| 30001(4x) | 8.125 efghi | 32.113 abc | 56.527 b | 28.389 bcd |
| 93/0098 | 14.605 efghi | 29.539 abcd | 59.829 b | 28.178 bcd |
| 01/1273 | 20.548 defghi | 24.033 cd | 57.489 b | 27.978 bcd |
| 92/1043(3x) | 12.595 efghi | 32.149 abc | 47.743 b | 28.389 bcd |
| 01/1371 | 17.038 efghi | 25.633 cd | 45.466 b | 29.472 abcd |
| M94/0461 | 9.274 efghi | 30.521 abc | 47.451 b | 29.880 abcd |
| 92/0398 | 15.591 efghi | 32.690 abc | 55.083 b | 25.737 cd |
| 97/1149 | 11.533 efghi | 33.139 abc | 55.804 b | 27.622 bcd |
| 99/2123 | 11.846 efghi | 32.635 abc | 43.902 b | 28.697 bcd |
| 92B/00061 | 13.896 efghi | 34.938 abc | 46.461 b | 27.526 bcd |
| 94/0039 | 12.860 efghi | 32.586 abc | 38.995 b | 29.132 bcd |
| 92/0057 | 11.676 efghi | 36.695 abc | 42.311 b | 28.274 bcd |
| 94/0020 | 12.381 efghi | 33.220 abc | 43.063 b | 28.397 bcd |
| 01/0265 | 4.202 i | 37.671 ab | 25.102 b | 29.713 abcd |
| 30001 | 13.995 efghi | 30.367 abc | 39.039 b | 30.040 abcd |
| 96/0867 | 7.265 efghi | 32.849 abc | 46.669 b | 29.020 bcd |
| W1095-D | 14.243 efghi | 30.597 abc | 44.056 b | 28.993 bcd |
| mm96/3665 | 12.967 efghi | 32.102 abc | 47.041 b | 28.389 bcd |
| 91/01730(4x) | 20.852 defghi | 24.062 cd | 48.467 b | 28.389 bcd |
| Tokunbo | 12.373 efghi | 32.514 abc | 74.630 ab | 26.360 cd |
| 01/1206 | 11.780 efghi | 30.452 abc | 44.933 b | 29.593 abcd |
| 93/0614(3X) | 13.014 efghi | 33.496 abc | 56.706 b | 26.580 cd |
| 95/0063 | 5.246 hi | 36.068 abc | 36.691 b | 29.312 bcd |
| 30572(CHK) | 14.841 efghi | 31.516 abc | 34.350 b | 29.371 bcd |
| 84/00136(4X) | 20.905 defghi | 22.915 cd | 37.373 b | 29.989 abcd |
| 93/0647(4X) | 22.119 defghi | 28.776 abcd | 27.949 b | 29.485 abcd |
| M98/0028 | 16.657 efghi | 31.690 abc | 56.000 b | 26.286 cd |
| M94/0177 | 9.869 efghi | 34.030 abc | 40.562 b | 28.855 bcd |
| Z95/0961 | 18.745 defghi | 29.545 abcd | 56.823 b | 26.990 bcd |
| 99/6017 | 17.639 defghi | 34.175 abc | 44.049 b | 26.901 bcd |
| 92B/0068 | 26.542 defgh | 28.135 bcd | 40.845 b | 28.389 bcd |
| 95/0180 | 20.300 defghi | 31.724 abc | 46.255 b | 27.315 bcd |
| 92/0427(4X) | 21.665 defghi | 27.454 cd | 55.060 b | 27.234 bcd |
| 96/0529 | 21.659 defghi | 29.027 abcd | 50.687 b | 27.193 bcd |
| 96/0565 | 6.810 efghi | 34.919 abc | 53.905 b | 27.477 bcd |
| 91B/00462 | 7.036 efghi | 35.260 abc | 41.842 b | 28.549 bcd |
| 92/0342(4x) | 14.944 efghi | 29.934 abcd | 46.947 b | 28.389 bcd |
| 96/1087 | 11.348 efghi | 30.951 abc | 44.361 b | 29.174 bcd |
| 01/1404 | 22.073 defghi | 30.235 abc | 42.403 b | 28.064 bcd |
| 91/1730(4X) | 13.715 efghi | 25.887 cd | 42.426 b | 31.107 abc |
| 91/00143(3X0 | 15.458 efghi | 35.973 abc | 9.925 b | 28.240 bcd |
| 91/01730 | 18.083 defghi | 27.848 bcd | 72.433 b | 26.545 cd |
| 95/0947 | 16.066 efghi | 31.899 abc | 49.738 b | 26.706 cd |
| MM97/0516 | 19.979 defghi | 31.241 abc | 42.230 b | 28.026 bcd |
| 96/1030 | 4.113 i | 33.496 abc | 42.473 b | 29.045 bcd |
| 98/0378 | 15.982 efghi | 32.163 abc | 47.714 b | 27.184 bcd |
| 97/0103 | 17.647 defghi | 29.639 abcd | 51.697 b | 27.130 bcd |
| bagi-wawa(4x) | 13.211 efghi | 31.454 abc | 45.164 b | 28.389 bcd |
| 98/0581 | 12.081 efghi | 31.485 abc | 69.079 b | 26.806 cd |
| 96/0037 | 0.719 i | 36.004 abc | 39.189 b | 28.453 bcd |
| 99/0411 | 15.897 efghi | 31.106 abc | 46.873 b | 27.901 bcd |
| 94/0459 | 17.635 defghi | 29.203 abcd | 39.573 b | 28.656 bcd |
| 91/02327 | 21.019 defghi | 33.014 abc | 29.458 b | 27.777 bcd |
| 89/02831 | 14.214 efghi | 27.689 bcd | 49.168 b | 28.389 bcd |
| Z95/0633 | 15.093 efghi | 27.465 cd | 65.981 b | 27.533 bcd |
| 01/1610 | 16.160 efghi | 24.995 cd | 39.037 b | 29.806 abcd |
| 97/0358 | 18.569 defghi | 32.197 abc | 42.134 b | 27.536 bcd |
| 95/0289 | 15.468 efghi | 29.930 abcd | 46.704 b | 28.322 bcd |
| 96/0903 | 10.797 efghi | 24.939 cd | 49.490 b | 29.248 bcd |
| 96/1165 | 14.093 efghi | 33.779 abc | 38.251 b | 28.161 bcd |
| 92/1029(3X) | 15.109 efghi | 30.321 abc | 57.003 b | 26.739 cd |
| 91/02327(4x) | 17.296 efghi | 28.615 bcd | 44.792 b | 28.389 bcd |
| 93/0279 | 22.767 defghi | 26.456 cd | 50.958 b | 26.638 cd |
| 94/0561 | 21.700 defghi | 26.610 cd | 51.343 b | 26.898 bcd |
| 95/0061 | 13.295 efghi | 33.594 abc | 44.179 b | 27.706 bcd |
| 96/1613 | 16.499 efghi | 29.807 abcd | 53.862 b | 26.824 bcd |
| 95/0680 | 6.188 fghi | 34.922 abc | 44.751 b | 28.369 bcd |
| 92/0326(4x) | 10.355 efghi | 30.397 abc | 49.187 b | 28.389 bcd |
| M94/0483 | 19.159 defghi | 17.261 d | 87.660 ab | 25.341 cd |
| 96/1427 | 9.744 efghi | 34.298 abc | 43.138 b | 28.209 bcd |
| 84/00316(4x) | 15.452 efghi | 21.467 cd | 48.741 b | 28.389 bcd |
| 4(2)1425 | 11.773 efghi | 32.804 abc | 36.229 b | 28.404 bcd |
| 95/0971 | 17.092 efghi | 28.534 bcd | 41.170 b | 28.411 bcd |
| 99/0446 | 17.249 efghi | 22.333 cd | 37.951 b | 29.359 bcd |
| 92/0326 | 14.341 efghi | 28.926 abcd | 61.404 b | 26.991 bcd |
| 99/1590 | 27.335 de | 27.443 cd | 36.010 b | 28.322 bcd |
| 96/0062 | 10.345 efghi | 30.924 abc | 38.450 b | 29.413 abcd |
| 4(2)1425(4x) | 15.426 efghi | 29.083 abcd | 44.160 b | 28.389 bcd |
| 01/0085 | 12.318 efghi | 32.306 abc | 44.488 b | 28.048 bcd |
| 99/0114 | 16.848 efghi | 32.123 abc | 42.636 b | 27.351 bcd |
| 99/0110 | 29.860 cde | 28.644 bcd | 33.842 b | 28.038 bcd |
| 89/00693 | 11.013 efghi | 28.161 bcd | 50.577 b | 28.389 bcd |
| 93/0665(3X) | 28.378 cde | 30.015 abc | 21.815 b | 27.967 bcd |
| MM97/0016 | 9.610 efghi | 26.839 cd | 68.938 b | 28.249 bcd |
| 99/0240 | 13.141 efghi | 31.194 abc | 36.118 b | 28.658 bcd |
| 97/1228 | 9.380 efghi | 29.590 abcd | 57.327 b | 28.108 bcd |
| 93/0267 | 9.338 efghi | 32.500 abc | 54.188 b | 26.785 cd |
| 01/0134 | 4.290 i | 33.156 abc | 35.153 b | 29.122 bcd |
| 88/00188 | 17.128 efghi | 23.467 cd | 45.626 b | 28.389 bcd |
| 96/0869 | 8.050 efghi | 31.563 abc | 37.067 b | 29.515 abcd |
| 01/1412 | 10.929 efghi | 25.591 cd | 41.070 b | 33.810 a |
| 92/0067(4X) | 18.166 defghi | 31.368 abc | 46.803 b | 25.261 cd |
| 94/0270 | 11.344 efghi | 36.189 abc | 42.932 b | 26.744 cd |
| 99/0564 | 10.624 efghi | 31.249 abc | 56.720 b | 26.982 bcd |
| 82/00058 | 14.635 efghi | 30.622 abc | 46.863 b | 27.584 bcd |
| 97/0296 | 11.648 efghi | 33.048 abc | 46.014 b | 27.331 bcd |
| 91/00255(3X) | 13.935 efghi | 36.843 abc | 30.567 b | 26.804 cd |
| 90/01554 | 13.184 efghi | 30.682 abc | 21.505 b | 29.206 bcd |
| 00/0340 | 8.508 efghi | 35.493 abc | 36.233 b | 28.236 bcd |
| 99/0543 | 13.115 efghi | 29.527 abcd | 42.319 b | 28.435 bcd |
| 92/0057(4x) | 9.871 efghi | 29.117 abcd | 48.800 b | 28.389 bcd |
| BEN86052(4X) | 12.939 efghi | 27.647 bcd | 47.113 b | 28.389 bcd |
| 4(2)1425(4X) | 14.685 efghi | 32.044 abc | 33.139 b | 28.326 bcd |
| M98/0034 | 13.675 efghi | 27.550 bcd | 56.296 b | 27.390 bcd |
| Z97/0474 | 24.151 defghi | 29.593 abcd | 29.826 b | 27.902 bcd |
| 01/1663 | 10.559 efghi | 26.271 cd | 61.848 b | 27.986 bcd |
| 96/0569 | 17.103 efghi | 26.623 cd | 56.320 b | 25.935 cd |
| 87/00018-4(4X) | 11.677 efghi | 33.833 abc | 42.426 b | 27.527 bcd |
| 96/0610 | 9.458 efghi | 32.257 abc | 47.194 b | 27.811 bcd |
| 91/00082(3X) | 15.311 efghi | 33.297 abc | 32.985 b | 27.581 bcd |
| 91/00143 | 2.183 i | 32.604 abc | 30.625 b | 29.289 bcd |
| Z96/0058 | 16.221 efghi | 33.806 abc | 38.250 b | 26.029 cd |
| 81/01635 | 18.485 defghi | 31.375 abc | 34.142 b | 27.793 bcd |
| Z97/0207 | 20.927 defghi | 32.182 abc | 24.029 b | 27.302 bcd |
| 91/02325(4x) | 9.718 efghi | 29.378 abcd | 47.718 b | 28.389 bcd |
| 91/02312 | 8.633 efghi | 37.005 abc | 38.230 b | 27.521 bcd |
| 91934(4x) | 13.609 efghi | 25.555 cd | 46.509 b | 28.389 bcd |
| 93/0589(3X) | 9.570 efghi | 30.984 abc | 13.820 b | 30.009 abcd |
| K95/0671 | 16.094 efghi | 32.461 abc | 29.117 b | 27.834 bcd |
| 91/00061(3X) | 11.526 efghi | 29.009 abcd | 57.363 b | 27.337 bcd |
| 93/0134 | 8.286 efghi | 32.874 abc | 50.317 b | 26.060 cd |
| MM96/4496 | 11.168 efghi | 29.112 abcd | 37.538 b | 29.335 bcd |
| Z98/0135 | 16.418 efghi | 24.360 cd | 56.297 b | 26.478 cd |
| 92/1154 | -0.065 i | 31.968 abc | 28.242 b | 29.601 abcd |
| 91/02316(4x) | 8.225 efghi | 29.065 abcd | 48.151 b | 28.389 bcd |
| 98/0002 | 9.349 efghi | 29.801 abcd | 43.412 b | 28.485 bcd |
| Abbey Ife | 9.832 efghi | 30.108 abc | 58.976 b | 26.633 cd |
| 92/0397 | 11.874 efghi | 32.048 abc | 47.801 b | 26.309 cd |
| 91/02316 | 7.725 efghi | 31.243 abc | 34.439 b | 29.115 bcd |
| 01/1380 | 15.293 efghi | 29.976 abc | 38.287 b | 28.182 bcd |
| 95/0166 | 9.798 efghi | 32.512 abc | 47.489 b | 26.679 cd |
| 99/0115 | 18.329 defghi | 21.447 cd | 42.426 b | 28.193 bcd |
| 93/0223 | 9.219 efghi | 29.943 abc | 36.819 b | 29.133 bcd |
| Alice local | 8.926 efghi | 36.664 abc | 39.623 b | 26.510 cd |
| 86/1(4x) | 12.848 efghi | 19.729 d | 46.449 b | 28.389 bcd |
| Z98/0187 | 14.614 efghi | 29.834 abcd | 41.105 b | 27.907 bcd |
| 96/1642 | 6.678 efghi | 23.895 cd | 49.287 b | 28.417 bcd |
| MS20 | 7.281 efghi | 29.519 abcd | 46.767 b | 28.389 bcd |
| 96/0557 | 6.100 fghi | 34.174 abc | 37.695 b | 28.072 bcd |
| 98/0196 | 10.237 efghi | 31.380 abc | 47.090 b | 27.309 bcd |
| 01/1086 | 12.649 efghi | 28.495 bcd | 30.132 b | 29.041 bcd |
| BAGI-WAWA | 11.690 efghi | 18.202 d | 46.885 b | 28.389 bcd |
| 95/0058 | 9.918 efghi | 29.531 abcd | 40.073 b | 28.419 bcd |
| 01/1115 | 9.290 efghi | 26.067 cd | 36.751 b | 29.784 abcd |
| Z95/0680 | 12.309 efghi | 25.440 cd | 54.919 b | 27.081 bcd |
| BEN 86052(4X) | 12.994 efghi | 25.929 cd | 25.898 b | 29.338 bcd |
| 93/0272 | 8.674 efghi | 25.673 cd | 53.157 b | 27.930 bcd |
| 92/0325 | 6.287 fghi | 34.609 abc | 41.012 b | 27.284 bcd |
| Z96/0038 | 10.351 efghi | 32.318 abc | 45.668 b | 26.155 cd |
| 90/02030 | 11.087 efghi | 25.562 cd | 38.558 b | 28.911 bcd |
| 99/7558 | 9.596 efghi | 29.171 abcd | 37.214 b | 28.668 bcd |
| 01/1442 | 10.981 efghi | 26.771 cd | 34.343 b | 29.049 bcd |
| 01/1235 | 13.412 efghi | 25.246 cd | 42.598 b | 28.305 bcd |
| 01/0171 | 8.677 efghi | 31.603 abc | 42.268 b | 27.936 bcd |
| 96/0963 | 7.911 efghi | 31.445 abc | 41.718 b | 28.189 bcd |
| 99/7559 | 5.578 ghi | 32.923 abc | 41.738 b | 27.658 bcd |
| 92/0067 | 12.181 efghi | 31.654 abc | 43.917 b | 26.438 cd |
| 97/0299 | 11.638 efghi | 29.078 abcd | 42.155 b | 28.288 bcd |
| 93/0639(3X) | 17.798 defghi | 29.671 abcd | 40.811 b | 25.685 cd |
| 91/00090 | 10.171 efghi | 24.496 cd | 14.724 b | 30.244 abcd |
| Z98/0139 | 11.858 efghi | 30.385 abc | 44.877 b | 27.068 bcd |
| 99/0222 | 13.614 efghi | 27.434 cd | 43.780 b | 27.708 bcd |
| 91/02325 | 7.146 efghi | 32.197 abc | 31.272 b | 28.383 bcd |
| ALICE LOCAL | 14.614 efghi | 28.540 bcd | 30.808 b | 28.283 bcd |
| 01/1443 | 7.661 efghi | 25.858 cd | 22.059 b | 30.238 abcd |
| 93/0271 | 12.738 efghi | 30.654 abc | 34.340 b | 27.818 bcd |
| 91/00419 | 12.327 efghi | 29.988 abc | 45.707 b | 26.301 cd |
| 93/0658 | 1.153 i | 37.496 ab | 23.094 b | 26.959 bcd |
| 99/0304 | 13.360 efghi | 28.938 abcd | 45.113 b | 26.783 cd |
| OFEGE | 13.387 efghi | 30.607 abc | 38.898 b | 27.014 bcd |
| 91/02325(4X) | 7.253 efghi | 31.011 abc | 23.494 b | 28.397 bcd |
| MS 20 | 11.414 efghi | 32.046 abc | 42.375 b | 25.830 cd |
| Z98/0190 | 20.661 defghi | 25.774 cd | 40.148 b | 24.999 d |
| M94/0121 | 13.034 efghi | 31.638 abc | 37.899 b | 26.330 cd |
| 93/0598(3X) | 19.739 defghi | 29.421 abcd | 23.263 b | 26.822 bcd |
| 91/00459 | 9.882 efghi | 29.822 abcd | 46.399 b | 26.963 bcd |
| Z92/0250 | 7.050 efghi | 24.453 cd | 41.683 b | 28.589 bcd |
| 91/02316(4X) | 8.293 efghi | 24.401 cd | 26.464 b | 29.524 abcd |
| 91/02317 | 6.989 efghi | 32.216 abc | 34.118 b | 27.888 bcd |
| 92/1043(3X) | 11.846 efghi | 30.783 abc | 31.350 b | 27.840 bcd |
| MM96/1751 | 16.968 efghi | 29.512 abcd | 38.410 b | 24.707 d |
| Z95/0594 | 8.312 efghi | 29.361 abcd | 29.626 b | 28.475 bcd |
| 99/0565 | 14.582 efghi | 31.966 abc | 27.268 b | 24.953 d |
| 01/1181 | 10.643 efghi | 31.377 abc | 38.706 b | 27.152 bcd |
| Z98/0193 | 9.915 efghi | 26.607 cd | 49.674 b | 23.964 d |
| 90/00330 | 8.930 efghi | 28.685 abcd | 44.279 b | 27.447 bcd |
| 91/02319 | 6.456 fghi | 31.067 abc | 33.276 b | 27.958 bcd |
| 95/0428 | 1.201 i | 32.347 abc | 29.291 b | 27.655 bcd |
| 99/0558 | 6.644 fghi | 30.460 abc | 36.429 b | 27.904 bcd |
| 01/1277 | 8.673 efghi | 26.917 cd | 43.803 b | 27.784 bcd |
| Ofege | 7.287 efghi | 32.077 abc | 19.198 b | 27.765 bcd |
| K93/0131 | 12.923 efghi | 27.094 cd | 36.062 b | 27.548 bcd |
| 97/4013 | 12.322 efghi | 28.312 bcd | 39.798 b | 26.806 cd |
| 91/00153 | 0.386 i | 33.835 abc | 14.060 b | 26.642 cd |
| Z95/0442 | 12.115 efghi | 28.598 bcd | 28.936 b | 27.430 bcd |
| 98/2101 | 5.516 hi | 30.579 abc | 40.219 b | 26.550 cd |
| 91B/00455 | 6.749 efghi | 30.858 abc | 32.804 b | 27.269 bcd |
| Z95/0098 | 3.840 i | 31.505 abc | 31.967 b | 26.689 cd |
| Z93/0151 | 7.584 efghi | 29.343 abcd | 32.889 b | 27.368 bcd |
| 95/0925 | 6.111 fghi | 29.579 abcd | 33.052 b | 27.186 bcd |
| Z95/0402 | 7.462 efghi | 29.828 abcd | 34.975 b | 26.247 cd |
| Z97/0219 | 7.823 efghi | 28.328 bcd | 30.724 b | 27.525 bcd |
| Z98/0137 | 13.358 efghi | 20.309 d | 27.469 b | 26.549 cd |
| Z94/0033 | 7.436 efghi | 30.036 abc | 17.457 b | 26.777 cd |
| Z96/0012 | 6.354 fghi | 19.495 d | 40.026 b | 27.028 bcd |
| 88/112-7 | 2.011 i | 22.004 cd | 8.113 b | 28.122 bcd |
| 99/2124 | 6.488 fghi | 29.592 abcd | 3.190 b | 25.865 cd |
| 88/112-7(3X) | 7.911 efghi | 20.312 cd | 15.993 b | 27.455 bcd |
| Z98/0044 | 8.650 efghi | 28.072 bcd | 13.063 b | 23.743 d |
| 91/02320 | 1.376 i | 28.008 bcd | 15.781 b | 26.580 cd |
| Pr > F(Clone) | <0.0001 | <0.0001 | <0.0001 | <0.0001 |
